# Relationship of Neutrophil-to-Lymphocyte Ratio with Carotid Plaque Vulnerability and Occurrence of Vulnerable Carotid Plaque in Patients with Acute Ischemic Stroke

**DOI:** 10.1155/2021/6894623

**Published:** 2021-06-21

**Authors:** Xin Li, Jing Li, Guode Wu

**Affiliations:** Department of Neurology, Lanzhou University Second Hospital, Lanzhou, 730000 Gansu Province, China

## Abstract

**Background:**

Carotid plaque is an undefined risk factor in ischemic stroke and is driven by inflammation. Mounting evidence suggests that neutrophil-to-lymphocyte ratio (NLR) is crucial not only for cerebrovascular events but also in atherosclerosis progression. Here, we aimed to explore the association between the admission NLR and carotid plaque vulnerability as well as the occurrence of vulnerable carotid plaque detected by carotid ultrasonography in patients with acute ischemic stroke (AIS) among Chinese.

**Methods:**

We conducted a retrospective study composed of 588 patients with AIS and 309 healthy controls free of carotid plaque in the Department of Neurology in The Second Hospital of Lanzhou University from March 2014 to February 2015. All patients were classified as nonplaque, stable plaque, and vulnerable plaque groups on the basis of carotid ultrasonography results. The baseline information was collected and compared among the four different groups. The correlation between variables and carotid plaque vulnerability was tested by Spearman linear correlation analysis. To identify the independent predictors for vulnerable carotid plaque, univariate and multivariate logistic regression analysis was performed.

**Results:**

The comparisons of age, sex proportion, history of hypertension, diabetes, and smoking, the levels of HDL-C, Lp(a), BMI, SBP, DBP, Fib, CRP, leukocyte, and NLR among the four groups showed a statistically significant difference (*P* < 0.05); in particular, the NLR was significantly higher in the vulnerable plaque group as compared to the control (*P* = 0.043), nonplaque (*P* = 0.022), and stable plaque groups (*P* = 0.015). The Spearman correlation analysis presented a positive correlation between carotid plaque vulnerability and age (*r* = 0.302; *P* < 0.001), SBP (*r* = 0.163; *P* < 0.001), and NLR (*r* = 0.087; *P* = 0.034), while the lymphocyte was negatively related to the carotid plaque vulnerability (*r* = −0.089; *P* = 0.030). The multivariate logistic regression analysis adjusted for confounding factors revealed that age (odds ratio [OR], 1.042; 95% confidence interval [CI], 1.025-1.060; *P* < 0.001), male gender (OR, 2.005; 95% CI, 1.394-2.884; *P* < 0.001), diabetes (OR, 1.481; 95% CI, 1.021-2.149; *P* = 0.039), SBP (OR, 1.012; 95% CI, 1.003-1.021; *P* = 0.010), and NLR (OR, 1.098; 95% CI, 1.018-1.184; *P* = 0.015) are independent predictors of vulnerable carotid plaque in patients with AIS.

**Conclusion:**

The admission NLR is a novel and meaningful biomarker that can be used in predicting carotid plaque vulnerability and the presence of vulnerable carotid plaque assessed by carotid ultrasonography in patients with AIS among Chinese.

## 1. Introduction

Stroke affects 33 million individuals worldwide annually, of whom 87% are ischemic [1]. The burden of stroke is greater in Asian countries than in the Western world, which is attributed to the higher incidence of stroke than coronary heart disease in Asians [2]. In China, ischemic stroke is a leading cause of mortality and disability and is projected to increase year by year. Atherosclerosis is the important pathologic basis of ischemic stroke and 30% of which results from carotid atherosclerotic disease [3]. Arterioarterial emboli caused by carotid vulnerable plaque rupture, ulceration, platelet activation, and thrombosis is considered to be the pathogenesis of ischemic stroke [4], and even the risk of vulnerable plaque rupture is more crucial than the severity of stenosis in ischemic stroke. For this reason, compared to patients with a stable 70% asymptomatic stenosis, the patient who has a low-grade stenosis but with an ulcerated plaque may benefit more from a revascularization procedure [5]. Therefore, judging the vulnerability of carotid plaque accurately contributes to stroke risk assessment and guides individualized treatment further, so as to prevent stroke effectively.

An increasing body of evidence suggests atherosclerosis is a concomitant inflammatory disease, which occurs firstly in the endothelium of the arterial wall [6]. As a subclinical sign of atherosclerosis, atherosclerotic plaque was found to hold various inflammatory cells, like activated T lymphocytes, macrophages, and mast cells [7]. What is more, it has been shown that circulating inflammatory markers including high-sensitivity C-reactive protein (hs-CRP), pentraxin 3 (PTX3), E-selectin, interleukin (IL)-6, tumor necrosis factor-alpha (TNF-*α*), and matrix metalloproteinases (MMPs)-9 are consistently associated with carotid plaque vulnerability [8, 9]. Thus, it is easy to see an apparent relevance regarding inflammation and plaque progression, and it is particularly critical to seek for sensitive biochemical indicators related to inflammation to distinguish the vulnerable carotid plaque.

As key elements of the immune system, leukocytes and their subtypes, neutrophils and lymphocytes, play a critical role in the immune response and inflammatory reaction. Neutrophil-to-lymphocyte ratio (NLR) can be readily calculated from the leukocytes related parameters, which combines neutrophils' nonspecific inflammation and lymphocytes' specific immune response into a single inflammatory biomarker. Previous investigations of NLR have reported that it can be used as a predictor of cancer [10, 11] and cardiovascular disease [12–15]. More recently, several studies have been carried out in cerebrovascular disease and shown strong predictive value of NLR for short-term prognosis in patients with AIS or transient ischemic attack [16–19]. However, the relationship between NLR and the evolution of carotid atherosclerotic plaque in patients with acute ischemic stroke (AIS) has been poorly understood to date.

Ultrasound is recognized to be an ideal tool for diagnosis and evaluation of atherosclerosis at present due to the advantages of noninvasive, convenient, and repeatability. Color Doppler ultrasound of carotid artery is applied to explore the information about the degree of stenosis, and the composition/surface of the carotid plaque, and to determine the pathology [20], has become the main imaging method for the examination of carotid plaque lesions. The purpose of this study was to investigate whether admission NLR is associated with carotid plaque vulnerability and the occurrence of vulnerable carotid plaque detected by carotid ultrasonography in patients with AIS among Chinese.

## 2. Methods

### 2.1. Study Population

We studied 588 consecutive AIS patients admitted to the Department of Neurology in The Second Hospital of Lanzhou University within seven days of stroke onset and underwent carotid ultrasonography imaging from March 2014 to February 2015. All the patients were in accordance with the Fourth National Stroke Conference revised cerebral infarction diagnostic standard, and the new ischemic lesions were confirmed by brain computed tomography (CT) and/or magnetic resonance imaging (MRI). Patients meeting any of the following criteria were excluded: (1) the patients who without a clear onset time, (2) patients with other cerebrovascular events rather than AIS, (3) patients who are suffering from serious cardiovascular diseases, blood disorders, and liver and kidney dysfunction; (4) patients who have severe infection, sepsis, malignant or autoimmune diseases, and who are taking immunosuppressants, glucocorticoid, or cytotoxic drugs; and (5) patients with missing data. The AIS patients were divided into nonplaque, stable plaque, and vulnerable plaque groups according to the carotid ultrasonography examination results below. In addition, another 309 healthy subjects without carotid plaque confirmed by carotid ultrasonography who were selected from the population simultaneously participated in a medical examination at the Center of Physical Examination in The Second Hospital of Lanzhou University were regarded as controls. The exclusion criteria of controls are the same as the AIS patients. Ultimately, a total of 588 consecutive AIS patients and 309 healthy subjects were recruited in this study.

The present study was approved by the Ethics Committee of the Second Hospital of Lanzhou University. Each participant was given a written and an oral description of the study and provided written informed consent before participating in this research.

### 2.2. Data Collection

A complete case history collecting, neurological examination, and demographic data recording were carried out by professional neurologists in all the patients within four hours after admission; electrocardiography (ECG), chest X-ray, brain CT or MRI, transcranial color-coded Doppler (TCD), and carotid ultrasonography were done in all the patients within 24 hours after admission. Clinical information was obtained from all patients, including age, sex, height, weight, the history of hyperlipidemia, hypertension, diabetes, coronary heart disease (CHD), atrial fibrillation (AF), smoking, and drinking. The body mass index (BMI) was calculated by dividing weight in kilograms by height in squared meters (Kg/m^2^). We also investigated the whole clinical data mentioned above and below of healthy subjects from their physical examination records.

### 2.3. Risk Factor Definitions

Hyperlipidemia was defined as total cholesterol (TC) ≥ 5.72 mmol/L and/or triglyceride ≥ 1.70 mmol/L, or use of lipid-lowering medications. Hypertension was described as systolic blood pressure (SBP) ≥ 140 mmHg and/or diastolic blood pressure (DBP) ≥ 90 mmHg, or a history of taking hypertension drugs. The diagnosis of diabetes depended on previous history of diabetes treated with or without antidiabetic agents or insulin, or fasting blood glucose (FBG) ≥ 7.00 mmol/L and/or 2 − hour postprandial blood glucose ≥ 11.10 mmol/L. The patients who with a typical clinical manifestation of CHD and confirmed before or a previous history of CHD were considered as CHD. The diagnosis of AF was based on the ECG manifestations. Smoking was determined as the average amount of cigarettes ≥ 10 per day and sustained more than one year. The patients who have an average amount of ethanol intake ≥ 30 g/d in the previous more than one year period were taken as drinking.

### 2.4. Laboratory Tests

The blood samples were taken from an antecubital vein after a 12-hour overnight fast and then stored at -80°C refrigerator before assay. Leukocyte counts and its different subtypes were determined by an automated blood cell counter (XE-2100, Sysmex, Kobe, Japan) according to the manufacturer's instructions, and NLR was calculated based on the results afterwards. An automatic biochemical analyzer (Cobas-8000, Roche, Basel, Switzerland) was used to measure many biochemical indexes, such as TC, triglyceride, high-density lipoprotein cholesterol (HDL-C), apolipoprotein AI (ApoAI), apolipoprotein B (ApoB), lipoprotein(a) [Lp(a)], fasting blood glucose (FBG.), homocysteine (Hcy), and C-reactive protein (CRP). The concentration of low-density lipoprotein cholesterol (LDL-C) was calculated as TC minus cholesterol in the supernatant by precipitation method. Fibrinogen (Fib) was quantified using the Beckman automatic coagulation analyzer (ACL-2000, Beckman Coulter, California, USA). SBP and DBP were measured at the right brachial artery of the subject using a standard mercury sphygmomanometer after five minutes of rest in the supine position.

### 2.5. Carotid Ultrasonography

The evaluation of carotid plaque was performed by using a color Doppler ultrasound with an 5 ~ 12 MHz linear-array probe (Philips-IU22, Royal Dutch Philips Electronics, Amsterdam, Holland) after a rest in the supine position for about 15 minutes. Putting a pillow below the subject's neck and making the head back with a slightly lateral rotation. Next, we scanned the bilateral common carotid artery, bifurcation, and internal and external carotid artery along the direction of vessel to determine the intima-media thickness (IMT). IMT was defined as the distance from the lumen-intima interface to the media-adventitia interface, and IMT > 1.5 mm protruding into the arterial lumen was regarded as carotid plaque formation, observing and recording the plaque characteristics including size, morphology, and property.

According to the morphological and acoustic characteristics of the plaques, they can be divided into (1) low-echo plaque: the fibrous cap is thin or unclear, and the interior is rich in lipid components, which is soft plaque, (2) medium-echo plaque: the fibrous cap is thick and mainly composed of collagen tissue, which is a fibrous flat plaque, (3) strong-echo plaque: the surface is smooth, but it can be accompanied by sound shadow in the rear, which is calcified hard plaque, and (4) mixed-echo plaque: the echoes vary in strength in more than 20% of the intraplaque area, suggesting that it is ulcerous plaque or mixed plaque with hemorrhage. Among them, medium-echo plaque and strong-echo plaque belong to stable plaques, while low-echo plaque and mixed-echo plaque were vulnerable plaques. Those with both stable plaques and vulnerable plaques were classified as vulnerable plaques.

### 2.6. Statistical Analysis

Baseline data of all participants is expressed as mean ± standard deviation (SD) for continuous variables and as percentages (proportions) for categorical variables. The Kolmogorov-Smirnov test was used to verify the normality of distribution of continuous variables. Multiple group comparisons of continuous variables were performed by one-way analysis of variance, followed by Tukey-Kramer posthoc analysis. The chi-square test was used to compare the categorical variables. The correlation between variables and carotid plaque vulnerability was tested by Spearman linear correlation analysis. To identify the independent predictors for vulnerable carotid plaque, multivariate logistic regression analysis was made by including the parameters that show significant values of <0.05 in univariate analysis, the odds ratios (OR) and 95% confidence intervals (CI) were calculated.

All statistical analyses were conducted by using SPSS for windows (version 19.0, Chicago, Illinois, USA). A two-tailed *P* value of <0.05 was considered as statistically significant.

## 3. Results

Our study population comprised 588 consecutive AIS patients and 309 healthy subjects totally. Of the 588 patients, 357 (60.71%) males, 231 (39.29%) females—with a mean age of 65.32 ± 11.43 years, 247(42.01%), 93(15.82%), and 248(42.18%) were in the non-, stable, and vulnerable plaque group, respectively. The mean age of 309 controls was 65.63 ± 10.63 years, and 53.72% of the subjects were male. The baseline characteristics of all study population are summarized in Table 1. The patients with vulnerable carotid plaque were obviously older and demonstrated higher prevalences of male gender, hypertension, diabetes, and smoking. Furthermore, they were more likely to present with increased Lp(a), BMI, SBP, DBP, Fib, CRP, and leukocyte and tended to have a lower HDL-C. The mean NLR of 588 patients was 2.86 ± 2.53, and the NLR was significantly higher in the vulnerable plaque group when compared to the control (*P* = 0.043), nonplaque (*P* = 0.022), and stable plaque groups (*P* = 0.015).

Table 2 shows the Spearman correlation analysis results of the associations between influence factors and the degree of carotid plaque vulnerability. Significant positive associations were observed for age (*r* = 0.302; *P* < 0.001), SBP (*r* = 0.163; *P* < 0.001), NLR (*r* = 0.087; *P* = 0.034), and carotid plaque vulnerability, and the age, SBP, and NLR increased progressively with aggravating of carotid plaque instability, whereas the lymphocyte was negatively related to the carotid plaque vulnerability(*r* = −0.089; *P* = 0.030), i.e., the lower level of lymphocyte and the poorer stability of carotid plaque (Table 2).

Univariate logistic regression analysis was performed for all the variables measured in this study. In order to determine the independent predictors of vulnerable carotid plaque, the variables found to be statistically significant in the univariate analysis were included in the multivariate logistic regression analysis. As a result, age (odds ratio [OR], 1.042; 95% confidence interval [CI], 1.025-1.060; *P* < 0.001), male gender (OR, 2.005; 95% CI, 1.394-2.884; *P* ≤ 0.001), diabetes (OR, 1.481; 95% CI, 1.021-2.149; *P* = 0.039), SBP (OR, 1.012; 95% CI, 1.003-1.021; *P* = 0.010), and NLR (OR, 1.098; 95% CI, 1.018-1.184; *P* = 0.015) were found to be the independent predictors of vulnerable carotid plaque (Table 3).

## 4. Discussion

In the current study, we demonstrated the relation between admission NLR with the extent of carotid plaque vulnerability and the presence of vulnerable carotid plaque evaluated by carotid ultrasonography in patients with AIS. The principal findings of this analysis were as follows: (1) there was an obvious trend of increased NLR levels in the vulnerable carotid plaque group compared to other groups, (2) NLR levels were positively correlated with the degree of carotid plaque vulnerability, and the stable carotid plaque was more likely to shift to vulnerable carotid plaque with the rise of NLR, and (3) elevated NLR significantly portended an increased risk of vulnerable carotid plaque in AIS patients, and the effect remained after adjusting the classical atherosclerotic risk factors, such as age, sex, diabetes, and blood pressure.

The pathogenesis of atherosclerosis is quite complex, the key processes of which involve vascular inflammation, lipid accumulation, intima thickening and fibrosis, arterial stiffness, remodeling, and plaque rupture or erosion [21]. The vulnerable plaque refers to that it is more prone to rupture and subsequently results in the distal embolization. Pathological characteristics of vulnerable plaque include a thin or ruptured fibrous cap, lipid-rich necrotic core, intraplaque hemorrhage, and intraplaque active inflammation [22]. Inflammation was recognized to be a key factor in influencing plaque vulnerability [23]. Besides, Biscetti et al. [24] found that proinflammatory genetic profiles were prominently more universal in subjects with vulnerable carotid plaque. Thus, we have reason to believe that inflammation biomarkers and proinflammatory cytokines are involved in the progression of carotid plaque and are closely related to plaque vulnerability. For example, Shindo et al. [8] studied 58 patients with carotid stenosis and observed that the levels of hs-CRP, PTX3, E-selectin, IL-6, and TNF-*α* were increased in the vulnerable plaque group compared with the stable plaque group; on the contrary, the anti-inflammatory cytokines IL-10 and adiponectin in the vulnerable plaque group were lower than those in the stable plaque group. However, as a marker of subclinical inflammation, the clinical relevance regarding NLR with carotid plaque vulnerability in patients with AIS has not been elucidated until now.

Neutrophil infiltration in the atherosclerotic plaque has been visualized immediately in vivo in animal models [25], and neutrophils mediate the inflammatory reaction by releasing numerous bioactive substances like arachidonic acid metabolites, platelet-aggravating factors, cytotoxic oxygen-derived free radicals, myeloperoxidase, elastase, and acid phosphatases, [12], leading to plaque evolvement and vulnerability gradually [26]; on the other hand, lymphocytes have been reported to be associated with the development of atherosclerosis [27, 28], and endogenous glucocorticoid secretes excessively in a stress state caused by inflammation, which brings about immunosuppression [29] and lymphocytes apoptosis [30]. Furthermore, the lymphocyte apoptosis tends to increase progressively with the intensification of atherosclerosis [31], while lymphocytes take part in anti-inflammation and endothelium protection. Hence, elevated NLR can objectively reflect systemic inflammation state because of the imbalance between neutrophils and lymphocytes, and the higher level of NLR suggests the more severe inflammation response [32]. In the present study, NLR was the highest in patients with vulnerable carotid plaque, and additionally, elevated NLR and decreasing lymphocyte were found to be correlated with exacerbation of carotid plaque vulnerability. As a consequence, NLR can express valuable information about the intergrated inflammatory activity of the vascular bed and reflects the degree of plaque burden in patients with atherosclerosis.

In a study which consisted of 399 coronary artery disease (CAD) patients with coronary lesions who underwent virtual histology-intravascular ultrasound, CAD patients with a raised NLR were observed to have more vulnerable plaque components [33], whereas Açar et al. [34] found that NLR was related to luminal stenosis rather than plauqe morphology in coronary artery. Our finding was compatible with the former, and we focused on patients with AIS and demonstrated that NLR but not neutrophil or lymphocyte even leukocyte appears to be a powerful predictor in determining the carotid plaque vulnerability in patients with AIS, which indicates a key role for NLR in the occurrence of vulnerable carotid plaque. A previous study reported that a higher NLR to be independently associated with arterial stiffness measured by brachial-ankle pulse wave velocity in the general population [35]. Lately, Hyun et al. [36] evaluated 252 cases of acute to subacute ischemic stroke assessing the carotid stenosis by carotid artery intima-media thickening (IMT), and they believed that NLR can be used to predict the degree of carotid stenosis in male patients with ischemic stroke. The results described above reveal a significant relationship between NLR and atherosclerosis collectively and prove that the inflammatory mechanisms are involved in the initiation and evolution of atherosclerosis as well as carotid plaques indeed. Therefore, our finding suggests NLR is more likely a risk factor than the differential leukocyte count being a predictor for vulnerable carotid plaque, independent of traditionary risk factors of atherosclerosis. In addition, Świtońska et al. [37] have reported that NLR at admission can accurately predict the risk of symptomatic hemorrhagic transformation in AIS patients undergoing revascularization. In a systematic review and meta-analysis published in 2017 [38], the authors confirmed that an elevated pretreatment NLR value is effective in predicting the risk of mortality or major adverse cardiac events in patients with a recent acute coronary syndrome. Therefore, NLR is expected to be an effective predictor for the prognosis of cardiovascular and cerebrovascular diseases.

Except for the results listed above, this research showed a positive correlation of carotid plaque vulnerability with age and SBP, as shown for NLR, age, male gender, diabetes, and SBP that are also found to be independent markers of vulnerable carotid plaque in AIS diseases. It is widely known that age, sex, hypertension, and diabetes are acknowledged risk factors of atherosclerosis [39, 40]. It has been suggested that carotid plaque morphology characteristics like plaque hemorrhage or thin fibrous caps vary with age and differ in sex [41]; Matsumoto et al. [42] observed that thicker and stiffer carotid arteries are more common in patients with type 2 diabetes than in the general population; moreover, hypertensive was reported to have a significantly higher carotid stiffness rate when compared to normotensive individuals [43]. Based on correlational clinical investigations aforementioned, we may speculate that various risk factors influence each stage of atherosclerotic progression process [44], for instance, IMT, plaque formation and stability shifting, carotid stenosis, or stiffness, in different manners.

To our knowledge, this is the first report examining the relationship between admission NLR and carotid plaque vulnerability as well as the occurrence of vulnerable carotid plaque in patients with AIS. In addition, our findings are persuasive and easy to be accepted due to the fact that we also included important covariates including well-established atherosclerosis risk factors such as age, sex, hyperlipidemia, hypertension, diabetes, and smoking; various blood lipid profiles, blood pressure, and glucose level; and kinds of serum index, for example, Hcy, Fib, CRP, and different leukocyte subtypes all that can reflect the systemic inflammatory information of the body to a variable extent. Adjustment for these potential confounders in multivariate models did not weaken the power of NLR in predicting vulnerable carotid plaque. The last but not the least, unlike other inflammatory markers such as hs-CRP, IL-6, TNF-*α*, and MMP-9, NLR can be readily and quickly obtained from blood routine text except cheapness. Meanwhile, it also conduces to assess the degree of carotid plaque vulnerability and stroke risk.

There are several limitations that should be taken into consideration. First, this is a retrospective and single-center study without a big enough sample size, which suggests that further large-scale randomized controlled trials are required to confirm the causality and generalize to all populations. Second, the property of carotid plaque was assessed by carotid ultrasonography, which has a limitation to detect individual plaque components and characteristics in detail on account of low sensitivity and specificity compared with MRI and CT [45]. Furthermore, NLR is a dynamic and time-varying marker result of the neutrophil and lymphocyte counts change over time. Beyond that, the level of NLR is likely to be affected by the body's own metabolism and external environmental factors, and so it would be particularly important to conduct the continuous follow-up ofthe NLR value, and we are on the point of performing it. Finally, despite the emerging evidence including our findings for the contribution of NLR to the atherosclerosis, the concrete mechanism of the influence of NLR on the atherosclerotic plaque remains to be clarified.

It is worth noting that the hemodynamic damage caused by carotid atherosclerosis is also the pathological mechanism of stroke, and inflammatory factors are also involved in this process. In recent years, vascular reactivity and cognitive dysfunction in patients with severe internal carotid artery stenosis after carotid endarterectomy have been investigated and discussed, and the association between the two has been explored [46, 47]. After the carotid revascularization, the diameter of the local stenosis can be significantly improved, so that the blood flow is unobstructed, the perfusion of the intracranial blood is increased, the reserve capacity of cerebral vascular is improved, the hemodynamics tends to be stable, and inflammatory factors may have corresponding changes in this process. Therefore, the correlation between NLR and cerebrovascular hemodynamic status and vascular reactivity will be the target of our further study.

In conclusion, our findings support the hypotheses that NLR can serve as a potentially useful indicator of the degree of carotid plaque vulnerability and that a higher NLR may be a clinically modifiable predictor of vulnerable carotid plaque among patients with AIS. The determination of NLR in patients with carotid plaque helps to identify those who are at risk and may benefit from vascular health management in clinical practice and also provides reference for stroke risk stratification and etiological prevention, as well.

## 5. Conclusion

The NLR is a novel and meaningful biomarker that is independently associated with the carotid plaque vulnerability and the presence of vulnerable carotid plaque detected by carotid ultrasonography in patients with AIS in Chinese.

## Figures and Tables

**Table 1:** Baseline characteristics of all study population in different groups.

Item	Control (*n* = 309)	Non-plaque (*n* = 247)	Stable plaque (*n* = 93)	Vulnerable plaque (*n* = 248)	*χ* ^2^/*F* value	*P* value
Age, years	65.63 ± 10.63	60.72 ± 12.03^a^	69.74 ± 9.70^ab^	68.25 ± 9.71^ab^	26.752	<0.001
Male, *n* (%)	166 (53.7)	138 (55.9)	50 (53.8)	169 (68.1)^abc^	13.939	0.003
Hyperlipidemia, *n* (%)	67 (21.7)	63 (25.5)	22 (23.7)	61 (24.6)	1.249	0.741
Hypertension, *n* (%)	195 (63.1)	176 (71.3)^a^	75 (80.6)^a^	200 (80.6)^ab^	24.842	<0.001
Diabetes, *n* (%)	64 (20.7)	61 (24.7)	27 (29.0)	87 (35.1)^ab^	15.345	0.002
CHD, *n* (%)	12 (3.9)	13 (5.3)	4 (4.3)	21 (8.5)^a^	5.955	0.114
AF, *n* (%)	8 (2.6)	17 (6.9)^a^	5 (5.4)	14 (5.6)	5.960	0.114
Smoking, *n* (%)	28 (9.1)	37 (15.0)^a^	21 (22.6)^a^	43 (17.3)^a^	14.013	0.003
Drinking, *n* (%)	21 (6.8)	28 (11.3)	14 (15.1)^a^	30 (12.1)^a^	7.486	0.058
TC, mmol/L	4.10 ± 1.01	3.86 ± 0.89^a^	4.20 ± 2.38^b^	4.00 ± 1.12	2.491	0.059
TG, mmol/L	1.41 ± 0.88	1.46 ± 0.93	1.56 ± 1.25	1.47 ± 0.85	0.683	0.563
HDL-C, mmol/L	1.28 ± 0.66	1.12 ± 0.29^a^	1.19 ± 0.33	1.16 ± 0.57^a^	5.178	0.001
LDL-C, mmol/L	2.57 ± 0.85	2.41 ± 0.85^a^	2.39 ± 0.83	2.50 ± 0.79	2.242	0.082
ApoAI, g/L	1.16 ± 0.29	1.08 ± 0.23^a^	1.17 ± 0.32^b^	1.07 ± 0.24^ac^	8.024	<0.001
ApoB, g/L	0.79 ± 0.21	0.76 ± 0.21^a^	0.77 ± 0.19	0.79 ± 0.22	1.635	0.180
Lp(a), nmol/L	34.09 ± 26.47	36.64 ± 27.67	44.41 ± 46.96^a^	41.07 ± 39.31^a^	3.405	0.017
BMI, kg/m^2^	20.40 ± 3.10	21.70 ± 3.18^a^	21.65 ± 3.20^a^	22.15 ± 3.13^a^	16.038	<0.001
SBP, mmHg	136.44 ± 17.60	136.53 ± 19.62	141.61 ± 18.23^ab^	143.55 ± 19.87^ab^	8.694	<0.001
DBP, mmHg	78.93 ± 11.04	81.05 ± 11.74^a^	80.52 ± 9.37	81.95 ± 12.08^a^	3.498	0.015
FBG, mmol/L	5.37 ± 2.06	5.75 ± 2.77	5.81 ± 2.43	5.79 ± 2.15^a^	2.026	0.109
Hcy, *μ*mol/L	20.91 ± 12.35	23.10 ± 24.24	22.66 ± 16.37	24.00 ± 16.45^a^	1.499	0.213
Fib, g/L	2.88 ± 0.64	3.04 ± 0.65^a^	3.11 ± 0.54^a^	3.06 ± 0.60^a^	6.270	<0.001
CRP, mg/L	21.31 ± 10.43	17.55 ± 12.27^a^	19.18 ± 17.08	19.80 ± 14.43	3.931	0.008
Leukocyte (×10^9^/L)	6.05 ± 2.00	6.66 ± 3.02^a^	6.32 ± 1.99	6.71 ± 2.55^a^	4.237	0.006
Neutrophil (×10^9^/L)	4.05 ± 1.75	4.19 ± 1.96	3.91 ± 1.36	4.24 ± 2.26	0.910	0.435
Lymphocyte (×10^9^/L)	1.67 ± 0.59	1.79 ± 0.61^a^	1.74 ± 0.49	1.67 ± 0.59^b^	2.406	0.066
NLR	2.77 ± 1.93	2.69 ± 1.97	2.48 ± 1.43	3.17 ± 3.22^abc^	2.837	0.037

Abbreviations: CHD: coronary heart disease; AF: atrial fibrillation; TC: total cholesterol; TG: triglyceride; HDL-C: high-density lipoprotein cholesterol; LDL-C: low-density lipoprotein cholesterol; ApoAI: apolipoprotein AI; ApoB: apolipoprotein B; Lp(a): lipoprotein(a); BMI: body mass index; SBP: systolic blood pressure; DBP: diastolic blood pressure; FBG: fasting blood glucose; Hcy: homocysteine; Fib: fibrinogen; CRP: C-reactive protein; NLR: neutrophil-to-lymphocyte ratio. ^a^*P* < 0.05, as compared to control; ^b^*P* < 0.05, as compared to nonplaque; ^c^*P* < 0.05, as compared to stable plaque.

**Table 2:** Correlation of influence factors and the degree of carotid plaque vulnerability.

Variables	*r* value	*P* value
Age	0.302	<0.001
SBP	0.163	<0.001
Lymphocyte	-0.089	0.030
NLR	0.087	0.034

Abbreviations: SBP: systolic blood pressure; NLR: neutrophil-to-lymphocyte ratio.

**Table 3:** Univariate/multivariate logistic regression analysis to determine the independent predictors of vulnerable carotid plaque.

Variables	Univariate			Multivariate		
	OR	95% CI	*P* value	OR	95% CI	*P* value
Age	1.042	1.026-1.058	<0.001	1.042	1.025-1.060	<0.001
Male gender	1.730	1.228-2.435	0.002	2.005	1.394-2.884	<0.001
Diabetes	1.547	1.084-2.210	0.016	1.481	1.021-2.149	0.039
SBP	1.015	1.006-1.023	0.001	1.012	1.003-1.021	0.010
Lymphocyte	0.740	0.556-0.984	0.038	0.968	0.676-1.387	0.861
NLR	1.091	1.017-1.171	0.015	1.098	1.018-1.184	0.015

Abbreviations: SBP: systolic blood pressure; NLR: neutrophil-to-lymphocyte ratio.
